# Reduction of liver macrophage transduction by pseudotyping lentiviral vectors with a fusion envelope from *Autographa californica *GP64 and Sendai virus F2 domain

**DOI:** 10.1186/1472-6750-9-85

**Published:** 2009-10-07

**Authors:** David M Markusic, Niek P van Til, Johan K Hiralall, Ronald PJ Oude Elferink, Jurgen Seppen

**Affiliations:** 1 AMC Liver Center, Amsterdam, The Netherlands

## Abstract

**Background:**

Lentiviral vectors are well suited for gene therapy because they can mediate long-term expression in both dividing and nondividing cells. However, lentiviral vectors seem less suitable for liver gene therapy because systemically administered lentiviral vectors are preferentially sequestered by liver macrophages. This results in a reduction of available virus and might also increase the immune response to the vector and vector products.

Reduction of macrophage sequestration is therefore essential for efficient lentiviral liver gene therapy.

**Results:**

Fusions were made of *Autographa californica *GP64 and the hepatocyte specific Sendai Virus envelope proteins. Lentiviral vectors were produced with either wild type GP64, Sendai-GP64, or both wild type GP64 and Sendai-GP64 and tested *in vitro *and *in vivo *for hepatocyte and macrophage gene transfer.

Sendai-GP64 pseudotyped vectors showed specific gene transfer to HepG2 hepatoma cells, with no detectable transduction of HeLa cervical carcinoma cells, and a decreased affinity for RAW mouse macrophages. Co-expression of wild type GP64 and Sendai-GP64 resulted in improved viral titers while retaining increased affinity for HepG2 cells.

In vivo, the Sendai-GP64 vectors also showed decreased transduction of murine liver macrophages.

**Conclusion:**

We demonstrate reduced macrophage transduction *in vitro *and *in vivo *with GP64/Sendai chimeric envelope proteins.

## Background

HIV-1 derived lentiviral vectors efficiently transfer genes and mediate long-term gene expression in non-dividing cells [1-3]. These properties make lentiviral vectors attractive candidates for the correction of inherited liver disorders. Lentiviral vectors pseudotyped with the envelope glycoprotein from Vesicular Stomatitis Virus (VSVg) can efficiently transduce primary hepatocytes *in vitro *[4,5]. In contrast, *in vivo *lentiviral vector delivery in rodents results in relatively poor gene transfer to hepatocytes because nonparenchymal liver cells are preferentially transduced [6-8]. We have previously shown that the main target of VSVg pseudotyped lentiviral vectors are liver macrophages, the Kupffer cells [9]. Although depletion of Kupffer cells leads to a significant increase in the gene transfer to hepatocytes [9] it would be preferable to develop lentiviral vectors that are less efficiently sequestered by macrophages. This would make more virus available for hepatocyte transduction and could also reduce the immune response to the viral vector and vector products.

Lentiviral vectors are commonly pseudotyped with VSVg which generates stable virions capable of transducing a broad range of cells both *in vitro *[10] and *in vivo *[10,11]. However, because this broad transduction range is not desireable in many applications, the use of alternative envelopes is investigated. The envelope protein from the baculovirus *Autographica californica *multiple nuclear polyhedrosisvirus, GP64, is also able to efficiently pseudotype lentiviral vectors [12]. GP64 pseudotyped lentiviral vectors exhibit comparable tropism and viral titers as that of VSVg, but with reduced cellular toxicity [12]. A comparison of *in vivo *gene transfer of lentiviral vectors pseudotyped with either VSVg or GP64 showed comparable transduction profiles in murine livers [13]. Thus, although earlier reports showed that baculovirus based vectors displayed a hepatocyte tropism[14,15], pseudotyping lentiviral vectors with the baculoviral GP64 protein does not appear to enhance hepatocyte gene transfer *in vivo*.

The engineering of retroviral envelope proteins for retargeting represent a challenge as modifications to viral envelope proteins often results in a significant reduction in viral titers [16-18]. GP64 seems to tolerate peptide insertions better than other viral envelopes and is therefore an attractive platform for the generation of targeted viruses. In baculovirus, amino terminal fusions to the GP64 envelope protein have been used for the surface display of; GFP [19], functional single chain antibody fragments [20], *Plasmodium berghei *circumsporozoite protein [21], avidin [22], and gp120 from HIV [23]. Lentiviral vectors can also be pseudotyped with engineered GP64 proteins, decay accelerating factor was fused to the amino terminus of GP64 and incorporated into lentiviral vector particles [24]. We have recently shown that GP64 can be used for the surface display of a peptide from the hepatitis B virus PreS1 protein resulting in pseudotyped lentiviral vectors with preferential gene transfer to liver derived cells [25].

The Sendai Virus Fusion (SV-F) protein utilizes a hepatocyte specific receptor for viral entry [26]. Both murine retroviral [27] and lentiviral vectors [28] could be pseudotyped with the SV-F protein, resulting in hepatocyte specific gene transfer, but viral particles were unstable and viral titers were too low to proceed to *in vivo *studies. We constructed a Sendai-GP64 fusion protein and investigated the tropism of lentiviral vectors pseudotyped with this envelope protein for hepatocytes and macrophages in vitro and in vivo.

## Results

### Construction of Sendai-GP64 chimeric envelope protein

**Figure 1:**
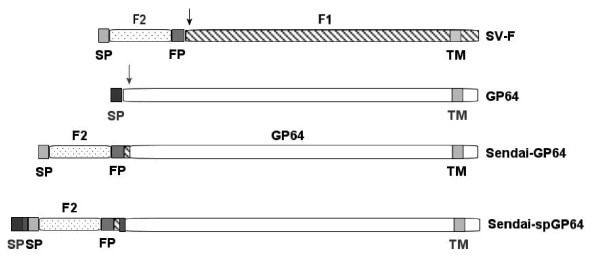
**Diagram of envelop constructs**. Linear map of the Sendai Virus Fusion (SV-F), wtGP64, Sendai-GP64, and Sendai-spGP64 glycoproteins with selected domains indicated: SP: Signal Peptide, FP: Fusion peptide, TM: Transmembrane.

The Sendai Virus Fusion protein (SV-F) is expressed as an inactive precursor protein F_0_, which is cleaved by a cellular protease to a F1 and F2 chain [29,30] (Figure. 1). A fragment containing the F2 domain and fusion peptide was fused to the amino terminus of GP64 (Figure 1). The resulting fusion gene of SV-F and GP64, Sendai-GP64, was verified by both restriction fragment analysis and sequencing.

**Figure 2:**
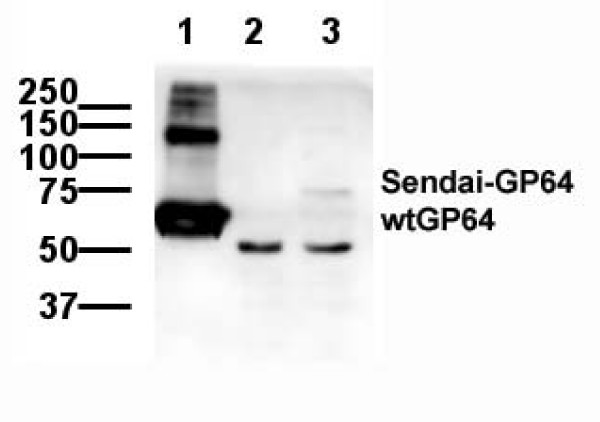
**Detection of GP64 proteins by western blot**. Concentrated viral vectors pseudotyped with wild type GP64, Sendai-GP64, and Sendai-spGP64 were subjected to western blotting and GP64 was detected using a monoclonal antibody. Lane 1 wtGP64, Lane 2 Sendai-GP64, Lane 3 Sendai-spGP64. The lane with wtGP64 pseudotyped virus shows strong reactivity at the expected size, 64 kDa. The higher molecular mass band likely represent GP64 trimers. The lane with Sendai-spGP64 pseudotyped virus shows reactivity at the expected size but at a much lower level. The lower molecular mass bands in the lanes with Sendai-GP64 and Sendai-spGP64 likely represent aspecific reactivity.

To investigate expression levels and incorporation into virus particles, Sendai-GP64 pseudotyped lentiviral vector particles were examined by western blotting. The monoclonal antibody (AcV5) directed against GP64 gave the expected band for the wild type GP64 pseudotyped lentiviral vectors (Figure 2 lane 1), but did not react with virus pseudotyped with Sendai-GP64 recombinant protein (Figure 2 lane 2). This was unexpected because Sendai-GP64 pseudotyped lentiviral vectors are capable of transducing cells and Sendai-GP64 contains the full length GP64 cDNA. However, the Sendai-GP64 fusion protein does not contain the native GP64 signal peptide and this may lead to differential posttranslational modifications abrogating binding of the AcV5 GP64 antibody. A new fusion protein was created where the Sendai Fusion F2 domain was inserted after the native signal peptide of GP64, Sendai-spGP64. Analysis of Sendai-spGP64 pseudotyped lentiviral particles on western blot produced a band migrating at the predicted size for the fusion protein (Figure 2 lane 3). However, compared to wtGP64, staining intensity of the Sendai-spGP64 protein was low. The identity of the low molecular mass band is unclear because it was not consistently present in western blots of concentrated GP64 virus. This lower band might therefore represent a degradation product of GP64, but could also be aspecific staining as more viral particles were loaded in the lane with Sendai-GP64 and Sendai-spGP64.

### Pseudotyping lentiviral vectors with Sendai-GP64 fusion proteins

Lentiviral vectors expressing GFP from the PGK promoter were produced with wtGP64, Sendai-GP64, or Sendai-spGP64 viral envelope proteins. Viral titers were determined on both HeLa (a human cervical carcinoma cell line) and HepG2 (a liver hepatoma cell line) cells. Lentiviral vectors pseudotyped with GP64 can efficiently transduce both HeLa and HepG2 cells, while both Sendai-GP64 and Sendai-spGP64 pseudotyped lentiviral vectors were only able to transduce HepG2 cells. Thus, both chimeric envelope vectors displayed a higher affinity for HepG2 cells as compared to wtGP64 (Table 1). Viral titers on HepG2 cells with Sendai-GP64 and Sendai-spGP64 vectors were approximately 2 orders of magnitude lower than those obtained with wild type GP64 (Table 1).

**Table 1:** Specificity of GP64 and GP64 variants for hepatoma cells and macrophages.

**Pseudotype**	**HeLa **	**HepG2 **	**RAW**	**Specificity** **HepG2 vs HeLa**	**Specificity** **RAW vs HeLa**	**Specificity** **HepG2 vs RAW **
wtGP64	4.2 × 10^5 ^± 3.1 × 10^5^	2.6 × 10^6 ^± 1.6 × 10^6^	4.0 × 10^5 ^± 3.2 × 10^5^	6.1	0.15	6.5
Sendai-GP64	UD^a^	2.7 × 10^4 ^± 1.3 × 10^4^	UD^a^	> 27	ND^b^	ND
Sendai-GP64/wtGP64	1.8 × 10^4 ^± 1.1 × 10^4^	5.1 × 10^5 ^± 2.7 × 10^5^	0.3 × 10^5 ^± 0.2 × 10^5^	28	0.06	17
Sendai-spGP64	UD^a^	1.2 × 10^4 ^± 0.4 × 10^4^	UD^a^	> 12	ND^b^	ND
Sendai-spGP64/wtGP64	2.2.10^4 ^± 0.5 × 10^4^	4.1 × 10^5 ^± 1.2 × 10^5^	2.3 × 10^5 ^± 1.3×10^5^	18	0.56	1.8

Lentiviral vectors were pseudotyped with: wild type GP64, Sendai-GP64 fusion protein, or generated with a mix of 29:1 of fusion- to wild type GP64 expression vector,, were used to transduce HeLa cervical carcinoma cells, HepG2 hepatoma cells and RAW macrophages.

Viral titers were determined using unconcentrated lentiviral vectors from at least two different virus preparations and are expressed as the amount of transducing units per ml. Specificity was calculated as the ratio of titers on HepG2 and HeLa cells and as the ratio of titers on RAW macrophages and HeLa cells.

^a ^Undetectable with detection limit of viral titers set to 1 × 10^3 ^TU/ml with flow cytometry. No GFP expressing cells were observed using a fluorescent microscope.

^b^Not determined.

The titers of lentiviral vectors with either the Sendai-GP64 or Sendai-spGP64 envelope alone were too low for *in vivo *use. Therefore, to increase viral titers, lentiviral vectors were produced with wtGP64 and either Sendai-GP64 or Sendai-spGP64 envelope proteins. The ratio of wtGP64 to Sendai-GP64 or Sendai-spGP64 plasmid used during virus production were optimised to yield lentiviral vectors that had high titers on HepG2 cells while retaining a reduced affinity for HeLa cells. The specificities of viruses produced with different ratio's of wild type GP64 and Sendai-GP64 are shown in table 2.

**Table 2:** Specificity of virus produced with different ratios of Sendai-GP64 to wild type GP64.

**Ratio**	**HeLa**	**HepG2**	**Specificity**
2:1	2.2 × 10^5 ^± 1.7 × 10^5^	1.6 × 10^6 ^± 1.2 × 10^6^	7.4
9:1	4.5 × 10^4 ^± 2.2 × 10^4^	7.1 × 10^5 ^± 2.1 × 10^5^	15.8
29:1	1.8 × 10^4 ^± 1.1 × 10^4^	5.1 × 10^5 ^± 2.7 × 10^5^	28.9
99:1	2.5.10^3 ^± 1.5 × 10^3^	7.7 × 10^4 ^± 1.6 × 10^4^	30.7

The ratio's 2:1, 9:1, 29:1, 99:1 refer to the relative amount of Sendai-GP64 to GP64 plasmid used for transfection during virus production. Viral titers were determined using unconcentrated lentiviral vectors from at least three different virus preparations.

We have previously shown that Kupffer cells (liver macrophages) are the predominant cell type transduced within the liver [9]. Therefore, it was important to determine the relative affinity of Sendai-GP64/wtGP64 and Sendai-spGP64/wtGP64 lentiviral vectors for macrophages. The mouse macrophage cell line RAW, was used as model to assess the relative gene transfer efficiency of our chimeric lentiviral vectors as compared to wtGP64. Wild type GP64 and Sendai-spGP64/wtGP64 pseudotyped lentiviral vectors were capable of gene transfer to this macrophage cell line, but titers were lower than those on HepG2 cells. However, Sendai-GP64/wtGP64 chimeric lentiviral vectors had even lower titers on RAW cells (Table 1), suggesting that Sendai-GP64 virus is detargeted from macrophages. The higher affinity for a hepatoma cell line (Table 1) and reduced gene transfer to macrophages (Table 1) suggests that only Sendai-GP64/wtGP64 and not Sendai-spGP64/wtGP64 lentiviral vectors would exhibit improved hepatocyte specificity *in vivo*.

### Significant reduction in gene transfer to nonparenchymal liver cells *in vivo *with Sendai-GP64/wtGP64 lentiviral vectors

**Figure 3:**
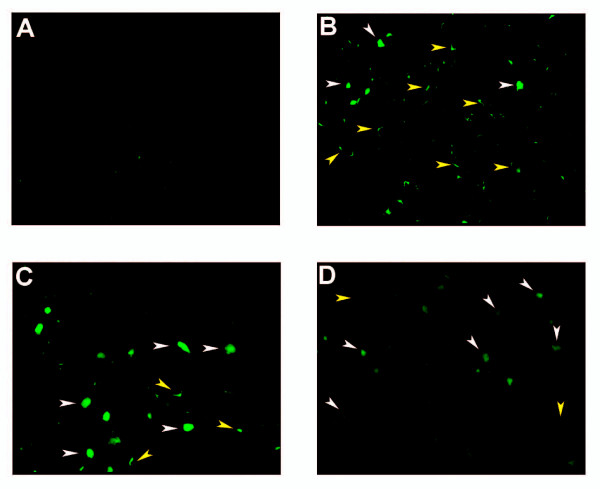
**Microscopic detection of GFP expression in the liver**. Expression of GFP in the liver following portal vein injection of either wtGP64, Sendai-GP64/wtGP64, or Sendai-spGP64/wtGP64 lentiviral vectors. Sections from the livers of (A) control (B) wild type GP64, (C) Sendai-GP64/wtGP64, (D) Sendai-spGP64/wtGP64 transduced mice. Low magnification (10×) fields are shown to give a representative view. White arrows indicate hepatocytes and yellow arrows indicate nonparenchymal liver cells. In wild type GP64 injected mice most GFP positive cells are nonparenchymal, in Sendai-GP64/wtGP64 injected mice the proportion of GFP positive nonparenchymal cells is much lower.

**Figure 4:**
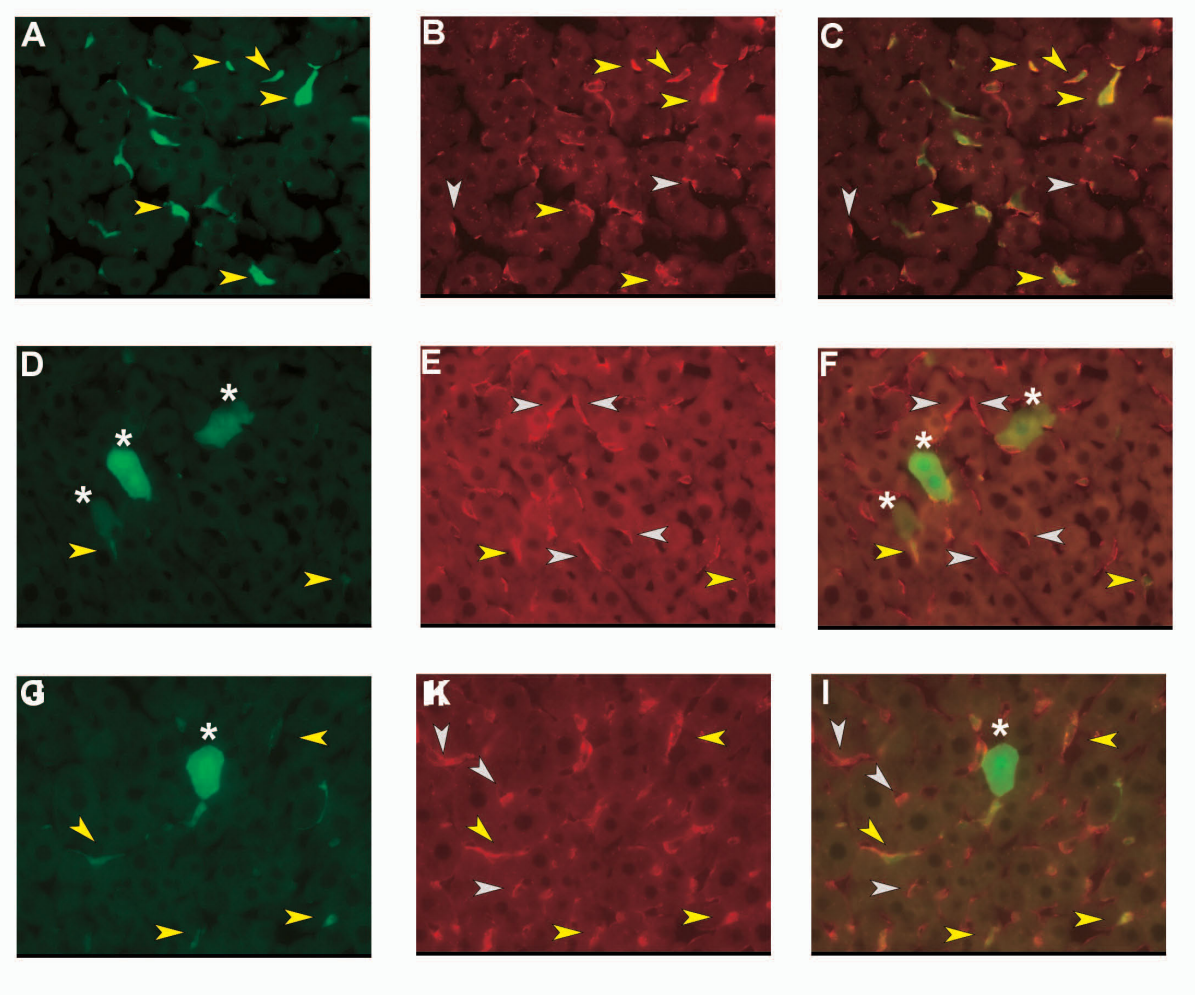
**Immunofluorescent staining of liver macrophages**. Liver sections were stained with an antibody against the F4/80 Kupffer cell marker. Kupffer cells are shown in red, GFP positive cell are shown in green. (A-C) Sections from a wtGP64 transduced mouse (A) GFP (B) Kupffer cells (C) composite. (D-F) Sections from a Sendai-GP64/wtGP64 transduced mouse (D) GFP (E) Kupffer cells (F) composite. (G-I) Sections from a Sendai-spGP64/wtGP64 transduced mouse (G) GFP (H) Kupffer cells (I) composite. Images are at 400× magnification. Yellow arrows indicate GFP positive Kupffer cells, grey arrows indicate GFP negative Kuppfer cells, and white stars denote GFP positive hepatocytes. In wild type GP64 and to a lesser extent Sendai-spGP64/wtGP64 injected mice many GFP positive Kupffer cells are observed. In Sendai-GP64/wtGP64 injected mice the level of Kupffer cell tranduction is much lower.

Male FVB mice 6 to 10 weeks old were injected intraportally with identical amounts of infectious virions, 0.5 × 10^8 ^HepG2 transducing units, of GP64 (6.3 μg HIV p24, n = 5), Sendai-GP64/wtGP64 (21.3 μg HIV p24, n = 7), or Sendai-spGP64/wtGP64 (4.6 μg HIV p24, n = 4) pseudotyped lentiviral vectors. One week following viral injections, the mice were sacrificed and tissues were fixed *in vivo*. Liver sections were prepared from the left and medial lobes and GFP expression was directly observed using fluorescence microscopy. In liver sections of mice injected with wtGP64 lentiviral vectors, the majority of transduced liver cells were nonparenchymal cells (Figure 3B), as previously described[13]. Staining of these sections with the F4/80 antibody confirmed that the majority of transduced non parenchymal cells are liver macrophages, the Kupffer cells. (Figures 4A-C). Strikingly, in the liver sections from Sendai-GP64/wtGP64 lentiviral vector injected mice but not in thet Sendai-spGP64/wtGP64 injected mice, the ratio of GFP expressing hepatocytes to nonparenchymal liver cells was much higher (Figures 4C and 4E).

Counting of GFP positive cells in liver sections showed a significant reduction (p < 0.005) in the amount of transduced nonparenchymal liver cells in mice injected with Sendai-GP64/wtGP64 lentiviral vectors as compared to wtGP64 (Table 3). Sendai-spGP64/wtGP64 injected mice had approximately two fold less gene transfer to hepatoctyes and liver nonparenchymal cells compared to wtGP64 treated mice (p < 0.05). The ratio of GFP positive hepatocytes to non-parenchymal liver cells of Sendai-GP64/wtGP64 pseudotyped lentiviral vectors is three fold increased as compared to both wtGP64 and Sendai-spGP64/wtGP64 (Table 3).

**Table 3:** Transduction efficiency of lentiviral vectors pseudotyped with GP64 variants on hepatocytes and non parenchymal cells of murine liver.

**Pseudotype**	**Hepatocytes/mm**^2^	**NPC/mm**^2^	**Specificity**
wtGP64 (n = 5)	9.9 ± 2.7	79.3 ± 12.1	0.125
Sendai-GP64/wtGP64 (n = 7)	9.5 ± 4.7	24.6 ± 19.6 ^a^	0.386
Sendai-spGP64/wtGP64 (n = 4)	5.6 ± 1.4^b^	47.5 ± 19.4^b^	0.118

Lentiviral vectors pseudotyped with wild type GP64 or mixes of wild type GP64 and Sendai-GP64 fusion proteins were generated and concentrated as described.

Mice were injected in the portal vein with 0.5 × 10^8 ^HepG2 transducing units of each virus. After one week frozen fixed liver sections were prepared from the medial and left lobes and GFP positive cells were counted using a fluorescence microscope.

NPC: nonparenchymal liver cells.

Specificity was calculated as the ratio of GFP positive hepatocytes/mm^2 ^to GFP positive NPC/mm^2 ^and indicates relative affinity for hepatocytes.

^a ^represents a significant difference (p < 0.005) between wtGP64 and Sendai-GP64/wtGP64 transduced animals. ^b ^represents a significant difference (p < 0.05) between wtGP64 and Sendai-spGP64/wtGP64 transduced animals.

Our observation that Sendai-GP64/wtGP64 pseudotyped lentiviral vectors have a reduced affinity for RAW macrophages in vitro is thus confirmed by the lower transduction of liver macrophages in vivo.

**Figure 5:**
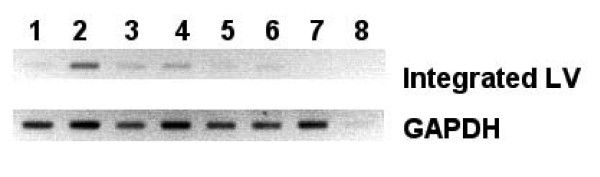
**PCR analysis of integrated vector genomes**. Genomic liver DNA from control mice and mice injected with different GP64 pseudotypes was isolated and PCR specific for integrated lentiviral vectors was performed. Lanes 1 and 2 wtGP64, 3 and 4 Sendai-GP64/wtGP64, 5 and 6 Sendai-spGP64/wtGP64, 7 control liver DNA, 8 water control. The strongest signals are seen in wild type GP64 transduced mice, confirming that these mice have more total GFP positive cells in the liver.

To validate the microscopy data, PCR amplification specific for integrated lentiviral vectors was performed on genomic DNA isolated from the liver. In the Sendai-GP64/wtGP64 and Sendai-spGP64/wtGP64 injected animals a weaker band is observed in liver genomic DNA, validating the results from the counting of GFP expressing cells in liver sections (Figure 5A lanes 3 and 4 versus 5 and 6). Unfortunately, transduction levels were too low for accurate quantitative PCR.

## Discussion

We report the first use of an amino terminal fusion to GP64 for the targeting of lentiviral vectors in the liver. The F2 domain of the Sendai virus fusion protein was fused to the amino terminus of GP64 and used to pseudotype lentiviral vectors. Lentiviral vectors pseudotyped with the Sendai-GP64 and Sendai-spGP64 fusion envelope were no longer able to transduce HeLa cells, but were still able to transduce HepG2 cells (Table 1). Viral titers were increased by co-production of both wild type GP64 and Sendai-GP64 (Table 2), in agreement with previous reports of GP64 fusion proteins both in baculoviral [23] and lentiviral vectors [24]. The presence of wild type GP64 may increase incorporation of Sendai-GP64 proteins into viral particles through a chaperone function or it may restore fusion activity that could be lost due to the addition of the SV-F2 domain [31].

Although Sendai-GP64 pseudotyped lentiviral vectors were capable of gene transfer, we were unable to detect expression of this chimeric fusion protein in viral particles and lysates of transfected cells (not shown) by western blot. This was surprising because the Sendai-GP64 construct contains the full length GP64 cDNA with exclusion of the signal peptide.

Lentiviral vectors produced without a viral envelope protein lack the ability to bind and fuse to target cells and are not capable of gene transfer in HepG2 cells under our experimental conditions (Markusic and Seppen, unpublished results). Thus, a functional envelope protein must be incorporated in the Sendai-GP64 pseudotyped virions. The most likely explanation for this discrepancy is that different posttranslational processing of Sendai-GP64 results in disruption of epitopes that are recognised by the antibody on wild type GP64.

Sendai-GP64 uses the Sendai virus signal peptide, when we constructed a Sendai-GP64 fusion protein that uses the GP64 signal peptide we were able to detect low levels of Sendai-spGP64 on western blot (Figure 2 lane 3) suggesting that the removal of the native GP64 signal peptide in Sendai-GP64 may effect protein processing and explain the loss of reactivity with the GP64 specific antibody AcV5.

Using the RAW mouse macrophage cell line as model for Kupffer cells, we showed that while wild type GP64 lentiviral vectors can efficiently transduce these cells, Sendai-GP64/wtGP64 lentiviral vectors transduce these macrophages with very low efficiency (Table 2). When administered *in *vivo, Sendai-GP64/wtGP64 lentiviral vectors exhibited a significant reduction of gene transfer to Kupffer cells (Table 3). Interestingly, Sendai-spGP64/wtGP64 pseudotyped lentiviral vectors transduced RAW mouse macrophages with a similar efficiency as wild type GP64 alone (Table 2) and this correlated well with increased gene transfer to Kupffer cells *in vivo *(Table 3). These data suggest that RAW mouse macrophages are a good model for assessing the gene transfer to liver Kupffer cells *in vivo*.

We evaluated three different pseudotyped lentiviral vectors (GP64, Sendai-GP64/wtGP64, and Sendai-spGP64/wtGP64) for *in vivo *gene transfer to hepatocytes in murine livers. Using GP64 pseudotyped lentiviral vectors as a reference, we observed a significant reduction in gene transfer to liver nonparenchymal cells with the Sendai-GP64/wtGP64 and Sendai-spGP64/wtGP64 lentiviral vectors (Table 3). Mice receiving Sendai-spGP64/wtGP64 lentiviral vectors had a two fold reduction in both hepatocyte and liver nonparenchymal cell gene transfer. The Sendai-spGP64/wtGP64 pseudotyped lentiviral vectors displayed a lower *in vitro *specificity for HepG2 cells (Table 1) and this in part may explain the lower gene transfer efficiency observed.

The titers of Sendai-GP64 pseudotyped lentiviral vectors are low and must be improved to be suitable for practical use. Inserting the Sendai fragment into a different site of GP64 or using a directed evolution approach might result in new and improved hybrid viral envelopes.

Hepatocyte specific gene transfer using GP64 pseudotyped Feline Immunodeficient Virus (FIV) derived lentiviral vectors has been described [32]. This hepatocyte specific gene transfer was not observed in our GP64 pseudotyped HIV derived lentiviral vectors (Figures 3B, 4C and Table 3) and in a previously published comparison of VSVg and GP64 pseudotyped HIV lentiviral vectors [13]. In both cases there is significant gene transfer to nonparenchymal liver cells. Differences in lentiviral vector systems and marker gene may explain these differences. In our hands, decreased nonparenchymal liver cell gene transfer was only observed with chimeric lentiviral vectors produced with wtGP64 and either Sendai-GP64 or Sendai-spGP64 (Figure 2C and Table 3).

## Conclusion

In this study we have shown that it is possible to redirect *in vivo *gene transfer through manipulations to the GP64 envelope protein. Further improvements in GP64 fusion proteins allowing for higher levels of expression may eventually lead to hybrid envelope proteins with complete retargeting without the need to co-express wild type GP64 envelope protein.

## Methods

### Construction of hybrid GP64 envelope proteins

The Sendai Virus Fusion cDNA was kindly provided by Dr. Allen Portner (St. Jude Children's Research Hospital) in a mammalian expression vector[33]. The GP64 cDNA was kindly provided by Marcel Westenberg, Wageningen University, and subsequently subcloned into the pCDNA 3.1p mammalian expression vector. An amino terminal truncation of GP64 was made by PCR to remove the native signal peptide (amino acids 1-21) retaining the native GP64 sequence starting at amino acid 25 using the following primers: ClaI GP64F 5'-GATCATCGATGAACGCGCAAATGAAGACGGGT-3' and GP64R 5'-TGCTGGATATCTGCAGAATT-3'. The resulting PCR product was cloned into the pCR2.1 TOPO TA vector (Invitrogen), pCR2.1 AA25GP64 and verified by sequencing. The Sendai-GP64 fusion construct was created by digestion of pCR2.1 AA25PG64 with ClaI and EcoRV to release a 1485 fragment containing the GP64 coding sequence. This fragment was cloned into a ClaI SmaI digest of the pCAG SV-F vector to create an in-frame F2-GP64 fusion cDNA. The resulting Sendai-GP64 fusion plasmid was verified by restriction digest and sequencing. A multiple cloning site containing a ClaI and AgeI restriction site was introduced immediately after the native GP64 signal peptide with the following primers as previously described [23].

MCSGP64for 5'GGTACCATGGTAAGCGCTATTGTTTTATATGTGCTTTTGGCGGCGGCGCA-3'

GP64midRev 5'-TAGATGCTGTTGTTGTAGC-3'.

The resulting PCR product was ligated into the pCR2.1 TOPO vector and sequenced. The GP64 coding sequence containing the MCS was released by a KpnI and SacII digest and subcloned into a KpnI and SacII digest of the pCDNA 3.1 GP64 to create pCDNA3.1 MCSGP64. The Sendai Virus F2 coding sequence was amplified with the following primers ClaISVF2f 5'-ATCGATATGACAGCATATATCCAGAGATC-3'

ClaISVF2r 5'-ATCGATTCTCGACTGGGGAGCACCGGCAT-3'

The resulting product was verified by sequencing and cloned into the ClaI site of MCSGP64 to create pCDNA 3.1 Sendai spGP64.

### SDS-PAGE and western blotting

Concentrated viral supernatants of GP64, Sendai-GP64/wtGP64, Sendai spGP64, and Sendai spGP64/wtGP64 pseudotyped lentiviral vectors were run on 10% SDS-PAGE and blotted onto nitrocellulose using the Bio-Rad Miniprotean III system. An antibody directed against a C terminal epitope in the GP64 protein, AcV5 (eBioscience 14-6995), was used at a 1:1000 dilution. A 1:1000 dilution of Goat anti Mouse HRP (Bio-Rad 170-6516) was used as a secondary antibody on all blots. Detection of reactive bands on western blots was performed using the Lumi-Light western Blot Substrate (Roche 12 015 200 001) and blots were analyzed using a LumiImager F1 and LumiAnalyst 3.1 software (Roche).

### Cell lines and culturing

HEK293T, HeLa, and HepG2 cells were grown in standard DMEM media supplemented with 10% fetal calf serum (FCS), 2 mM glutamine, 100 U/ml penicillin, and 100 μg/ml streptomycin at 37°C in 10% CO_2_. RAW mouse macrophages were grown in standard RPMI media supplemented with 10% fetal calf serum (FCS), 2 mM glutamine, 100 U/ml penicillin, and 100 μg/ml streptomycin at 37°C in 10% CO_2_

### Lentiviral vector production

Lentiviral vectors with the phosphoglycerate kinase promoter driving eGFP expression were produced as described earlier [4]. Briefly, lentiviral vectors were produced by transient transfection of 293T HEK cells using calcium phosphate precipitation. Sendai-GP64/wtGP64 pseudotyped lentiviral vectors were produced using 6.8 and 0.2 μg of plasmid per plate respectively. Sendai-spGP64 pseudotyped lentiviral vectors used 50 μg of envelope plasmid per plate and Sendai-spGP64/wtGP64 used 50 and 0.5 μg of envelope plasmid respectively. Viral supernatants were concentrated for animal experiments and western blotting by overnight centrifugation using a Hettich centrifuge (2230 g). Viral titers were determined by titration on both HeLa and HepG2 cells. Briefly transductions were performed for four hours (HeLa and HepG2) and overnight (RAW) in the presence of DEAE Dextran (10 μg/ml) and 72 hours later cells were analyzed by flow cytometry for GFP expression. GFP positive cells were not observed when viral transductions of HepG2 cells were performed in the presence of the HIV reverse transcriptase inhibitor AZT (Retrovir) indicating the absence of pseudotransduction.

Viral particles were measured using a commercial ELISA kit for the Gag p24 protein (Perkin Elmer NEK050). HepG2 titers for the concentrated lentiviral vectors ranged from 1.5 - 3.2 × 10^8 ^transducing units/ml and p24 levels ranged from 4-30 μg/ml.

### Animals, viral injections, and tissue processing

Wild-type FVB male mice ages 6-10 weeks were used in all studies and were fed *ad libitum *on standard laboratory chow. All animal experiments were performed in accordance with the Animal Ethical Committee guidelines at the Academic Medical Center of Amsterdam.

Mice were anesthetized with an intraperitoneal injection of FFM mix (2.5 mg/ml Fluanisone/0.105 mg Fentanyl citrate/1.25 mg Midozalam HCl/kg in H_2_O, 7 ml/kg). Under deep anesthesia, the peritoneal cavity was opened and the mice were injected intraportally with identical amounts of infectious virions, the equivalent of 0.5 × 10^8 ^HepG2 transducing units, on day 0. The amount of HIV p24 injected was 6.3 μg for GP64, (0.25 ml of 2.0*10^8 ^TU/ml n = 5), 21.3 μg for Sendai-GP64/wtGP64, (0.35 ml of 1.5*10^8^TU/ml) n = 7), and 4.6 μg for Sendai-spGP64/wtGP64, (0.16 ml, of 3.1*10^8^TU/ml) n = 4. The peritoneal cavity was sutured and the animals received the analgesic Temgesic (20-30 μl, 0.03 m mg/ml) subcutaneously following recovering from FFM.

On day 7, the mice were killed by *in vivo *fixation. Under deep anaesthesia, the peritoneal cavity was opened and a ligature was place around the anterior right lobe of the liver, tightened, and the lobe was excised and snap frozen in liquid nitrogen for genomic DNA analysis. Subsequently, the animals were perfused intracardially with 20 ml of phosphate buffered saline (PBS), followed by 20 ml of 2% formaldehyde in PBS. Following perfusion, the liver and spleen were removed and further fixed for 4 hours in 4% formaldehyde in PBS at room temperature. The fixed tissues were then transferred to 30% sucrose solution and incubated overnight at 4°C, snap frozen in liquid nitrogen and stored at -80°C the following day.

Cryosections were made from both the left and medial lobes. The tissue was embedded in Tissue-Tek OCT (Bayer) and sections (6 μm) were applied to poly lysine coated glass slides and enclosed in Vectashield mounting media (Vector Laboratories).

### Immunostaining

Liver sections were prepared as described above and frozen without mounting media. Following thawing at room temperature sections were washed three times 5 minutes each in PBS and blocked with 10% Normal Goat Serum in PBS/0.05% Tween-20 for one hour. Kupffer cells were stained with a rat anti mouse F4/80 antigen (1:20 Serotec) for one hour. Slides were washed three times 5 minutes each in PBS/0.05% Tween-20 and incubated with a goat anti rat Texas Red conjugated antibody (1:500 Rockland Immunochemicals) for one hour. Following three washing steps of 5 minutes each, sections were embedded in Vectashield mounting media containing DAPI. Images were captured at (400×) magnification using a fluorescent microscope (Leica DMRA2).

### Cell counting and statistics

GFP positive cells were counted in sections made from the left and median lobes using a fluorescent microscope (Leica DMRA2). All sections/slides were prepared and coded independent of the counter. Identification of parenchymal and nonparenchymal cells was made based on morphology as described before [9].

Per animal, one section from the left lobe and median lobe were counted for GFP positive hepatocytes at (200×). Three fields (200×) per section were counted for GFP positive nonparenchymal liver cells. Images of the counted sections were captured and the surface area of sections was calculated using Leica FW4000 software. The number of total hepatocytes per mm^2 ^was estimated by counting amount of hepatocytes present in one field at (400×). Data are reported as number of GFP expressing cells per square millimeter.

Statistical analysis was performed using SPSS 11.0 software using the Mann-Whitney U test. Values were determined to be significantly different with p < 0.05.

### Genomic DNA isolation and PCR

Genomic DNA was isolated from snap frozen liver and spleen tissue using Dneasy tissue kit (Qiagen) according to manufacturers instructions. The following primer pairs were used to generate a 274 bp product: HIV-U3 forward primer 5'-CTGGAAGGGCTAATTCACTC-3' and HIV PSI reverse primer 5'-GGTTTCCCTTTCGCTTTCAG-3'. This primer pair is designed to specifically amplify integrated provirus and thus reduce contamination from amplification of the transfer plasmid. Additionally primers directed against GAPDH were used as a template loading control GAPDH forward primer 5'-CAATCACCATCTTCCAGGAG-3' and GAPDH reverse primer 5'-TGCCCACAGCCTTGGCAGC-3'. 100 ng of total DNA was used per PCR reaction using the following conditions: 95°C for 5 minutes, followed by 33 cycles of 95°C for 30 seconds, 55°C for 30 seconds, and 72°C for 30 seconds with a fill in at 72°C for 10 minutes. Negative control samples were taken from animals that had not been injected with virus.

## Authors' contributions

DMM and JS conceived the study.

DMM, NPVT, JKH and JS designed and performed experiments.

DMM, RPJOE and JS analyzed the data.

DMM and JS wrote the manuscript.

All authors read and approved the manuscript.
